# Exploring psychosocial interventions for people with dementia that enhance personhood and relate to legacy- an integrative review

**DOI:** 10.1186/s12877-016-0250-1

**Published:** 2016-04-05

**Authors:** Bridget Johnston, Melanie Narayanasamy

**Affiliations:** Sue Ryder Care Centre for the Study of Supportive, Palliative and End of Life Care, School of Health Sciences, The University of Nottingham, Queen’s Medical Centre, Nottingham, NG7 2HA England, UK

**Keywords:** Dementia, Alzheimer’s disease, Cognitive impairment, Personhood, Legacy, Dignity, Integrative review

## Abstract

**Background:**

Epidemiological predictions suggest that dementia will continue to rise and that this will have social and economic ramifications. Effective interventions, beyond pharmacological management are needed. Psychosocial interventions have largely been investigated in relation to carers of people with dementia, or with regards to their ability to manage dementia symptoms, improve cognition, and reduce challenging behaviour. However, since dementia is a life-limiting illness and people with dementia are at risk of having their personhood compromised, psychosocial interventions should seek to enhance personhood, and offer the potential for the person to leave a legacy.

**Methods:**

An integrative review was carried out to identify, assess, appraise and synthesise studies featuring interventions, which relate to both personhood and legacy. Search strategies were developed in key databases: MEDLINE; PsycINFO; Embase; Joanna Briggs Institute; CINAHL; Cochrane Database of Systematic Reviews; ASSIA. Grey literature was also identified through free-text searches.

**Results:**

Thirty six articles were included in the final review, these were tabulated and were assessed based on how the intervention related to personhood and legacy. Classification resulted in three themes being identified: *Offering aspects of legacy; Acknowledging the person behind the patient; Facilitating meaningful engagement.* Generally, personhood aspects of interventions were well reported, but further research is required to explore legacy potential of psychosocial interventions for people with dementia.

**Conclusion:**

The integrative review provides an overview and exploration of an under-researched area, and provides directions for future research, which will help expand the evidence base and ultimately help improve patient care for people with dementia and their families.

## Background

Dementia affects almost 50 million people worldwide, manifesting as deterioration of cognitive functions, such as memory, thinking and behaviour (World Health Organisation/WHO). There are many types of dementia including vascular dementia, mild cognitive impairment and Alzheimer’s disease [1, 2]. Dementias have been identified as progressive, life-limiting illnesses resulting in complex needs [3], dementia can be a burden both socially, and economically, and has been regarded as a key health and public health priority [4, 5]. Epidemiological predictions suggest that dementia will continue to rise [3, 6, 7] with estimated figures suggesting that there will be 1.7 million people living with dementia in the UK by 2051 [8]. Therefore, social and economic ramifications can be expected to persist. This means that effective interventions and treatments, grounded in theoretical and empirical evidence bases, are required to manage dementias and reduce the social and economic burden for people. Dementia care and services have become more prominent on government agendas in recent years, with greater media coverage also capturing the public’s attention and awareness [9].

However, it is argued that care provision has failed to meet the complex needs affecting the growing proportion of people living with dementia [10]. It has been postulated that post-diagnostic support should be effective and holistic [4], focusing on enabling people with dementia to live well with their condition [11], and be committed to maintaining the person’s independence as much as possible, ultimately adhering to the ethos of person-centred care [8]. The pharmacological management of dementia has often involved addressing what the literature term “challenging behaviour” [12], with antipsychotic medication. This has had negative outcomes [10, 13]. Associated side effects of antipsychotic medications have been described as adverse [14] and particular medication has been found to be ineffective for certain aspects of dementia, such as agitation [12].

Therefore, there is increasing attention being given to incorporating non-pharmacological psychosocial interventions in dementia care [15–18], which can improve quality of life. These include life story work, reminiscence therapy, music therapy, approaches to interaction and communication, environmental modifications and reality orientation [8, 17]. The variety of psychosocial interventions that are available may help people with dementia to build coping strategies, reduce distress, provide interpersonal connections and optimise remaining abilities [18].

### Previous systematic reviews of psychosocial interventions for people with dementia

Nevertheless, previous systematic reviews on psychosocial interventions for people with dementia have neglected to give specific attention to how they may enhance personhood and/or offer the person an opportunity to leave a legacy. The majority of reviews have focused primarily on psychosocial interventions for caregivers of those with dementia to support them in their caregiving activities and enhance their wellbeing, rather than looking at the person with dementia [19–24]. Those which have addressed psychosocial interventions for people with dementia, have focused on the agenda to ameliorate symptoms [25–27], reduce challenging behaviours, (including agitation and wandering) [12, 28–32]; enable a reduction of medication [33], improve cognitive function [34, 35], or a combination of all of these [36]. Whilst important to the landscape of dementia research, such foci steers away from the areas this current review desires to investigate.

Lawrence and colleagues [37] explored psychosocial intervention benefits for people with dementia through qualitative evidence synthesis. This included identifying interventions’ ability to facilitate meaningful engagement and contributions. However, this work was mainly explored in the context of finding out how to best implement interventions into practice. Bates et al. [38] systematic review on psychosocial interventions for people with mild dementia identified reality orientation, procedural memory stimulation and counselling. Whilst the first two interventions were discussed in relation to their effect on mental health, the identified outcome measures for counselling were related to wellbeing. However, in addition to this only focusing on people with mild dementia, the review was conducted over a decade ago. In addition, Kasl-Godley and Gatz’s review [18] provided a useful overview of six different psychosocial interventions for people with dementia. The authors present both the theoretical background and empirical evidence of these interventions. The psychosocial interventions reviewed were psychodynamic approaches including psychotherapy; reminiscence and life review. The findings acknowledge that reminiscence allows interpersonal functions to be achieved such as leaving a legacy; support groups, recognising the applicability of these for the person with dementia as well as the people caring for them; reality orientation, which targets confusion amongst people with dementia; memory training, targeted at improving memory performance and memory functioning; and behavioural approaches, focusing on reducing what are perceived to be undesirable behaviour. No previous systematic reviews were found particularly concerned with life review.

Therefore, there is still sparse recent evidence offering specific focus on the aims we wish to address. We hypothesise that psychosocial interventions could do much more to promote personhood as well as serve a purpose of leaving a legacy of the person as they become more cognitively impaired. We argue that this should be focused on in evidence synthesis.

### Personhood and legacy

Person-centred care is endorsed as part of good health care practice and encompasses a holistic and personalised ethos, as well as, being part of conserving the dignity of the person [39–41]. Dignity-conserving care is highlighted as a necessary element of all health care and a responsibility for all healthcare professionals [39, 42]. The notion of “personhood” is inherently part of person-centredness [43], and is even more prominent in healthcare conditions involving dementia, which can challenge person-centredness [44]. Personhood denotes the elements of human beings that make them a person and is a status that is given by others, assuming recognition, respect and trust, [43–45]. Stein-Parbury et al. [46] argue that person-centred care for people with dementia is driven by the belief that it is possible to maintain personhood regardless of cognitive impairment. Furthermore, personhood is made up of personal, relational, existential and moral elements [47].

It is acknowledged that people with dementia may be at risk of having their personhood compromised [48]. Given this acknowledgment, this review will focus on identifying and appraising psychosocial interventions, which enhance personhood. Moreover, although not widely acknowledged, dementia is a life-limiting illness by nature of it significantly shortening the person’s life [49]. Thus, the review also seeks to explore the extent to which interventions offer the potential to allow the person to leave a legacy. The definition of legacy is:

1. *Law.* a gift of property, especially personal property, as money, by will; a bequest.
2. anything handed down from the past, as from an ancestor or predecessor [50].

The second definition informs our understanding of a legacy component to psychosocial interventions. Therefore, for the purposes of this review, leaving a legacy denotes situations in which the person with dementia can reveal and/or leave behind aspects of their personhood, for example their life story, identity, or insights into their former roles and achievements. With psychosocial interventions such as life story work, this results in a tangible object, such as a book [51] or memory box [52]. Since dementia can lead to erosion of personhood and aspects of identity being lost [47], psychosocial interventions which serve to facilitate and preserve personhood are welcomed. We postulate that the ability to leave a legacy can support enhancement of personhood. Therefore, interventions such as life story work involve reviewing a person’s past life and producing their individual biography [51]. This allows the personhood of the individual to be demonstrated and can give a sense of the ‘person behind the patient,’ this may involve making links between the person’s past and present [45]. This may help health and social care staff to respond more appropriately and sensitively to people’s needs, because they have a better-informed insight into the person.

## How and why the psychosocial interventions were chosen

An initial scoping stage navigating existing literature to help identify psychosocial interventions, ascertain their theoretical origins and empirical evidence-base, and assess their potential to help people with dementia symptoms retain and/or enhance their personhood, as well as, allow them to leave a legacy. The psychosocial interventions were identified through initial MEDLINE, Google Scholar and Google searches using the MeSH headings and free text terms around “psychosocial intervention”, “Dementia”, “Alzheimer’s Disease”, Mild Cognitive Impairment”, “Person-centred” and “Personhood” and appropriately combining them. Including “legacy” as a term was unsuccessful in returning articles featuring such psychosocial interventions. Therefore, consulting with experts (in person and via email) in the field was also necessary (in conjunction with searches) to help interventions of interest to be identified. These two methods allowed seven different “types” of psychosocial interventions to be identified all of which had clear theoretical origins. Biographical approaches included Life Story Work, Dignity Therapy, Reminiscence Therapy and personal profile documents, which all in some form or another apply narrative theory to healthcare. Each of these encourages individuals to reflect and disclose aspects of their life experiences [18, 53–55]. Doll therapy has its roots in a psychological approach linking to Bowlby’s [56] attachment theory and involves the person interacting with a doll or similar object [57]. Also stemming from psychological origins is person-centred counselling, influenced by humanistic psychology. This intervention sees the person have sessions with a trained therapist who uses attentive listening and empathy to help the person resolve problems [58]. Finally, creative therapies, such as art, music and drama therapy were identified, these engage the person in creative activities and are underpinned by both psychology and the psychiatric approach of moral treatment [59].

These seven interventions were then considered more critically by two authors (BJ and MN) based on the extent to which they related to both personhood and legacy in the context of the person having a dementia condition. This was undertaken by further searching the theoretical and empirical evidence around them. The decision was made to discount person-centred counselling, since the legacy potential could not clearly be established. Therefore, the search strategy was developed around the six remaining psychosocial interventions.

## Methods

### Aims and objectives

The integrative review identifies, appraises, selects and synthesises existing research on psychosocial interventions for people diagnosed with dementia. Specifically, it seeks to address the following questions:

- What is the evidence for effective psychosocial interventions used for people with dementia, which enhance their personhood and offers the potential for them to leave a legacy?
- What recommendations can be made for clinical practice?

Furthermore, the review was guided by the following aims:

1. To identify, select, appraise and synthesise available evidence regarding psychosocial interventions for people with dementia, which enhance personhood and allow the person to leave a legacy.
2. To compare and contrast the different psychosocial interventions based on their ability to enhance personhood and allow the person to leave a legacy.
3. To make health and social care recommendations regarding psychosocial interventions relating to legacy and enhancement of personhood for people with dementia.
4. To outline future research avenues to expand the evidence base regarding psychosocial interventions relating to legacy, which enhance personhood for people with dementia.

### Ethics

Since this was a literature review no ethical permissions or informed consent were needed. Any supporting data related to the review, not in the article can be obtained from the corresponding author.

### Search of the literature

Dementia symptoms were taken to include the different types of dementia conditions identified by UK charity Alzheimer’s Society, such as Alzheimer’s disease, vascular dementia, dementia with Lewy bodies, and mild cognitive impairment. In the identification stage, psychosocial interventions were chosen based on their potential to enhance personhood of the individual with the condition and allow them to leave a legacy.

**Fig. 1:**
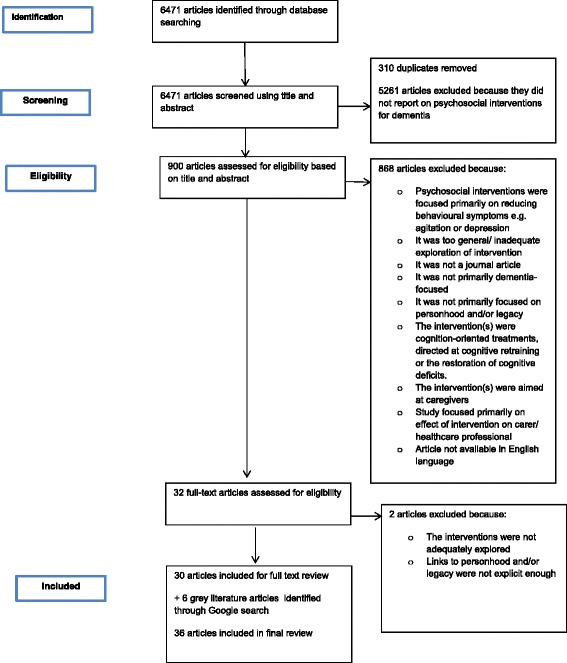
PRISMA flow diagram

The initial scoping stage was pivotal to allow a search strategy to be developed, which was modified appropriately for the key electronic databases. Please see Table 1 for the search strategy developed for Ovid MEDLINE. The databases consulted for this integrative review were: Ovid MEDLINE; Ovid PsycINFO; Ovid Embase; Ovid Joanna Briggs Institute; CINAHL; Cochrane Database of Systematic Reviews; and ProQuest ASSIA. Searches were carried out between February and March 2015 using a combination of Medical Subject Headings (MeSH)/ EMTREE key words and free text terms. Free text terms were largely set to be identified within the whole document, but were refined for some databases to be limited to title and abstract, if more appropriate. Figure 1 displays a “Preferred Reporting Items for Systematic Reviews and Meta-Analyse” (PRISMA) flow diagram which captures the phases of the integrative review undertaken to reach the final number of included articles. Guidance for producing the PRISMA diagram were gained from the PRISMA website and divide the process into four steps: “Identification”, “Screening”, “Eligibility” and “Included”.

**Table 1:** MEDLINE search strategy

Number	Search terms
1.	psychosocial intervention$.mp.
2.	psychosocial intervention$.mp.
3.	1 or 2
4.	non pharmacological intervention$.mp.
5.	life stor$.mp.
6.	story telling.mp.
7.	storytelling.mp.
8.	oral histor$.mp.
9.	biograph$.mp.
10.	exp personal narratives/
11.	narrative therapy/
12.	personhood/
13.	dignity therap$.mp.
14.	reminisc$ therap$.mp.
15.	doll therap$.mp.
16.	play therap$.mp.
17.	Play Therapy/
18.	exp animal assisted therapy/
19.	pet therap$.mp.
20.	writing therap$.mp.
21.	((poetry or poem$) adj3 therap$).mp.
22.	person centred counsel$.mp.
23.	rogerian.mp.
24.	1 or 2 or 3 or 4 or 5 or 6 or 7 or 8 or 9 or 10 or 11 or 12 or 13 or 14 or 15 or 16 or 17 or 18 or 19 or 20 or 21 or 22 or 23
25.	exp Dementia/
26.	exp Alzheimer Disease/
27.	Mild Cognitive Impairment/
28.	cognitiv$ impair$.mp.
29.	dementia.mp.
30.	alzheimer$.mp.
31.	25 or 26 or 27 or 28 or 29 or 30
32.	24 and 31

### Search outcomes

After search strategies were performed, 6471 articles were identified and retrieved. These underwent initial screening by one author (MN), based on title and abstract. This resulted in 5261 articles being excluded for not sufficiently focusing on psychosocial interventions in dementia. Articles were exported from host websites of selected databases to Endnote × 6 reference manager. This allowed a total of 310 duplicate articles to be identified and removed. This then led to the next stage of assessing eligibility, where two authors (BJ and MN) assessed the remaining articles based on title and abstract, against inclusion criteria (please see Table 2). This helped exclude a further 868 articles. Full texts were retrieved for these remaining articles, which were then assessed against the inclusion criteria. Two articles were excluded through this assessment, which led to 30 full text articles being included in the final review.

**Table 2:** Inclusion criteria

Inclusion criteria	Rationale
Literature published since 1990	Initial scoping indicated that some interventions had relevant literature from the 1990s. Accepting literature from 1990 onwards ensures that key empirical and theoretical evidence on relevant interventions are not missed.
Intervention(s) must be psychosocial in nature i.e., as according to the Oxford English Dictionary definition of psychosocial: “Of or relating to the interrelation of social factors and individual thought and behaviour” (OED, 2015).	The review is focused on psychosocial intervention(s) for people with dementia
Intervention(s) must be non-pharmacological	The review is not concerned with drug-related interventions and therefore focuses on non-pharmacological intervention(s) only
Intervention(s) must have the potential to enhance personhood and enable the person to leave a legacy	Personhood and legacy factors are major foci of this review.
Intervention(s) must be designed for human adults with dementia	The review is concerned with relevant psychosocial intervention(s) that are used on adults with dementia. For the purposes of this review, dementia is understood in accordance with the definition present on Alzheimer’s Society’s (2015) website: “a set of symptoms that may include memory loss and difficulties with thinking, problem-solving or language”.
Describes the results of empirical and theoretical research studies	This review is concerned with identifying, appraising, and synthesising best available evidence. As such empirical studies are deemed the strongest source of evidence-base. Theoretical studies are also included if evidence was deemed relevant.
Consults evidence from grey literature	Initial scoping suggested that some psychosocial interventions used for people with dementia have not have been theoretically and/or empirically investigated. Therefore, grey literature provides the best insight into these particular interventions. For the purposes of this review, grey literature is understood to be literature that has not formally been published in sources such as books or journal articles (as advised in the Cochrane Handbook, 2011)
English language	Budgetary constraints have meant that only English texts can be reviewed, to save on translation costs
An exclusively Western focus	The findings of this review will contribute to recommendations for health and social care practice and future research avenues. These will be made with Western settings in mind.

In addition to empirical and theoretical research studies, the grey literature was also consulted for relevant papers. Two authors (BJ and MN) used Google search engine searches to identify and assess the grey literature, against the inclusion criteria, which led to 6 sources of grey literature being included in the final review. Therefore, for the final review 36 articles/reports were included.

### Methodological and theoretical rigour of included articles

Sources of information were kept broadly open to allow thorough exploration of the full evidence base regarding our selected psychosocial interventions. The full spectrum of evidence was accepted, also, because personhood and legacy aspects have not been the foci of previous research regarding psychosocial interventions and dementia. However, some limits were placed to focus on databases renowned for returning results relevant to nursing, health and social care-related disciplines. This approach meant that a methodological assessment of articles was not included, since the nature of evidence pertinent to the review enquiry was found to be heterogeneous, with varied empirical and theoretical research identified. Nevertheless, as identified in the initial scoping stage, the theoretical basis of all included interventions was ascertained.

### Classifying previous research

The full text included articles were then read and analysed for themes which summarised the consistent ideas and patterns present in the data through the use of key words. Analysis was directed to focus on anything relevant to addressing the first research question and therefore centred on legacy and personhood. This has resulted in the themes relating significantly to aspects of legacy and personhood. Analysing the literature in this way enables conceptual constructs to be classified under specific themes, and helps to generate new knowledge. As a result, this review fulfils the aims of an integrative review [60]. Classifying literature evidence under themes also allows gaps and shortcomings to be apparent, thus helping to inform a future research agenda [39].

### Data synthesis

Through the process of classifying previous research, data from the articles were placed appropriately under the relevant developed theme. By presenting the data under themes, this enabled the data to be understood with regards to how the interventions offered the person with dementia the opportunity to leave a legacy and also how the interventions enhanced personhood.

## Results

**Fig. 2:**
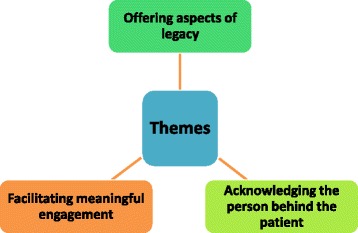
Themes generated from the included articles

The themes generated from the included articles are captured in Fig. 2 and are indicated for each study in Table 3 where relevant. These themes enable assessment of the interventions in terms of their ability to enhance personhood and offer the potential for legacy (a key aim of this review as indicated in the Methods section). *Offering aspects of legacy* captures examples where the psychosocial interventions within the included articles allow the person with dementia to leave a legacy through tangible or non-tangible means or the potential to do so. It was not always an explicit aim of the article to show or acknowledge how the intervention linked to legacy. Therefore, *offering aspects of legacy* is partly based on our (BJ and MN) recognition of the intervention’s legacy potential. *Acknowledging the person behind the patient* denotes ways in which the interventions enable aspects of the person to come to the fore beyond their illness. This includes their past and present roles and elements of identity. This was seen to contribute to insights into both legacy and personhood. Finally *Facilitating meaningful engagement* focuses on the person’s response to the intervention, with regards to participation, enjoyment and enhancement of personhood.

**Table 3:** Overview of included articles

Reference	Psychosocial intervention	Article type/Brief summary	Generated theme
1. Benbow B. (2014) Design features for resident engagement and meaningful activity, Canadian Nursing Home, 25(4): 4–8 [68]	- Reminiscence; Design features for example building lifestyle stations which will stimulate particular memories and influence increased engagement in activities	- Theoretical - Literature review of empirical and practice-based recommendations on designing residential environments, e.g., residential homes, that will facilitate meaningful activities. - De-emphasising dementia illness and deterring the view of people as passive recipients of care	*Acknowledging the person behind the patient* *Offering aspects of legacy* *Facilitating meaningful engagement*
2. Buse C. and Twigg J. (2014) Women with dementia and their handbags: Negotiating identity, privacy and “home” through material culture, Journal of Aging Studies, 30, 14–22 [69]	- Reminiscence (using clothing and handbags to stimulate memories and access to personal histories).	- Empirical - Part of ESRC funded UK study on Dementia and Dress. - Used observations and qualitative interviews. “Wardrobe interviews” were also conducted- interviewing people next to their wardrobes. - Sample 32 case studies with dementia (9 men and 23 women). -Female participants of different socio-economic backgrounds- 10 lived in their own homes; 13 in care homes. - Used reminiscence groups,based on idea that handbags are linked to memories and identities, reacquainting women with happier times.	*Acknowledging the person behind the patient* *Offering aspects of legacy*
3. Chaudhury H. (2003) Quality of Life and Place-Therapy, Journal of Housing For the ElderlyJournal of Housing For the Elderly, 17 (1-2_: 85–103 [67]	Place-Therapy; Reminiscence	- Empirical - Study exploring reminiscence of personally meaningful places from the past for nursing residents cognitively impaired and non-cognitively impaired). - Residents from four different nursing homes; Interviews with 13 residents with dementia, 15 family members of residents with dementia; and 8 residents with no cognitive impairments - Reminiscing encouraged by the narrative of lived experiences elicited from places. - Article describes place-therapy as a potential therapeutic intervention, but it would need to be implemented as an ongoing activity to allow better evaluation.	*Acknowledging the person behind the patient* *Offering aspects of legacy* *Facilitating meaningful engagement*
4. Chung JCC. (2009) An intergenerational reminiscence programme for older adults with early dementia and youth volunteers: values and challenges, Scandinavian Journal of Caring Sciences, 23, 259–264 [78]	Reminiscence therapy; Life story work	- Empirical Pre and post one group design was used; 49 older participants with early dementia and 117 youth volunteers from Hong Kong - Older participants were assigned to 2 youth participants and all took part in a 12-session reminiscence programme. - Youth participants were facilitators who also helped the older participants to create a personal life story book - Findings revealed that this intergenerational reminiscence programme had mutual benefits for both participants. - Findings were based mostly on feedback from youth participants around their opinions of the reminiscence programme. - More detailed analysis and discussion around gains for the older participants would be helpful	*Offering aspects of legacy*
5. Cohen GD. (2002) Familiar activities, videos can help patients cope with memory loss, Geriatrics, 57(3): 62-65 [61]	Life story work; video biographies	- Theoretical - Literature review looking at nonmedical interventions that can bring about patient satisfaction. - Video biographies are explored as a way of conveying the person’s life history to families, friends and volunteers and encourage these latter groups to visit.	*Offering aspects of legacy* *Facilitating meaningful engagement*
6. Cooney A., Hunter A., Murphy K., et al. (2014) “Seeing me through my memories”: a grounded theory study on using reminiscence with people with dementia living in long-term care, Journal of Clinical Nursing, 23, 3564–3574 [71]	Reminiscence	-Empirical - Grounded Theory using in-depth interviews with residents with dementia (*n* = 11), relatives (*n* = 5), healthcare assistants (*n* = 10), nurses (*n* = 9), and nurse managers (*n* = 3)- exploring their perceptions about reminiscence. - Study participants were recruited from long-term care facilities where reminiscence was being used. - The theory generated a theory “seeing me through my memories”, which highlights the way in which reminiscence and engaging with the patient allowed the staff to see the person and enhance personhood.	*Offering aspects of legacy* *Facilitating meaningful engagement*
7. Crete- Nishihata M., Baecker RM., Massimi M., et al. (2012) Reconstructing the Part: Personal Memory Technologies Are Not Just Personal and Not Just for Memory, Human-Computer Interaction, 27 (1–2): 92–123 [63]	Life story work- using personal memory technologies	- Empirical - Study of 12 participants with Alzheimer’s Disease (AD) or Mild Cognitive Impairment (MCI) and family members - DVD-based Multimedia Biographies (MBs) capturing events, people, and places from participants’ past. - MB content included photographs, home movies, documents, music and narration. Participants and family members contributed to content	*Acknowledging the person behind the patient* *Offering aspects of legacy* *Facilitating meaningful engagement*
8. Damianakis T., Crete-Nishihata MC., Smith KL., et al. (2009) The Psychosocial Impacts of Multimedia Biographies on Persons With Cognitive Impairments, The Gerontologist, 50 (1): 23–35 [64]	Life story work	- Empirical - Multimedia biographers and social workers conducted interviews with 12 family members of people with Alzheimer’s Disease (AD) and Mild Cognitive Impairment (MCI) to gain insight into patients’ life histories and build Multimedia Biographies (MBs). - Also collected were archival materials to contribute to life histories.	*Offering aspects of legacy* *Facilitating meaningful engagement* *Acknowledging the person behind the patient*
9. Dempsey L., Murphy K., Cooney A., et al. (2014) Reminiscence in dementia: A concept analysis, Dementia, 13(2): 176–192 [100]	Reminiscence Therapy	- Theoretical - Concept analysis; Literature review - Useful to define “reminiscence” so that an operational definition can be generated and to allow it to be developed in dementia care. - Beyond the concept analysis, the article offers some exploration of the use of reminiscence as a therapeutic intervention for people with dementia.	*Acknowledging the person behind the patient* *Facilitating meaningful engagement*
10. Fels DI. And Astell AJ. (2011) Storytelling as a Model of Conversation for People With Dementia and Caregivers, American Journal of Alzheimer’s Disease & Other Dementias, 26 (7): 535–541 [90]	Storytelling	- Empirical - Study applies a storytelling conventional model to verbal reminiscences of older people with dementia. - 27 older adults with dementia were recruited from a day care centre and social work department care home. - Used photographs of 6 different annual events (Christmas, Easter, Burns Night, New Year, Birthdays, Holidays). Participants were shown a series of 6 photographs and were encouraged to disclose memories of each event. The interviewer was able to guide participants where necessary.	*Offering aspects of legacy*
11. Hagens C., Beaman A., and Ryan EB. (2003) Reminiscing, Poetry Writing, and Remembering Boxes, Activities, Adaptation & Aging, 27(3–4): 97–112 [52]	Reminiscence; Poetry writing	-Empirical - Reminiscence sessions were carried out with 5 cognitively-impaired older adults. Their words and phrases were structured into poetry to convey their “essence”. - Information elicited from these session was used to build personal Remembering Boxes, containing meaningful objects and writings, - Participants were 5 nursing home residents (1 man; 4 women) who had some level of cognitive impairment. - 7 group sessions were conducted, lasting for about an hour, and tasking place is “casual” settings. These preceded or proceeded interviews, with the aim to further explore meaningful memories.	*Offering aspects of legacy* *Acknowledging the person behind the patient* *Facilitating meaningful engagement*
12. Gibb H., Morris CT., and Gleisberg J. (1997) A therapeutic programme for people with dementia, International Journal of Nursing Practice, 3, 191–199 [70]	Reminiscence	-Empirical - Reports on a trial programme incorporating Tai Chi and subsequent reminiscence sessions used on 9 people with moderately advanced dementia. - Analysis focuses on the stories told by the people and aims to understand the purpose of storytelliing for them. - Participants were 9 older residents of a nursing home. 56 % had multi-infarct dementia; 44 % had Alzheimer’s Disease. - 13 research sessions were conducted twice a week over 7 weeks.	*Offering aspects of legacy* *Acknowledging the person behind the patient*
13. Heathcote J. (2010) Paws for thought: involving animals in care, Nursing & Residential Care, 12(2): 145–148 [66]	Animal assisted intervention/Animal assisted therapy/Pet therapy	- Theoretical - literature review around the benefits of how animals can impact on residents in nursing homes and how pets can be used in therapy. - Cautions/ negative aspects are also explored - Article provides some guidance for staff who may want to bring a resident animal.	*Offering aspects of legacy* *Acknowledging the person behind the patient* *Facilitating meaningful engagement*
14. Heathcote J. and Clare M. (2014) Doll therapy: therapeutic or childish and inappropriate, Nursing & Residential Care, 16 (1): 22–26 [57]	Doll therapy	- Theoretical - Literature review exploring the benefits and controversial aspects of doll therapy on people with dementia. - Ethical issues are also addressed, e.g., whether it is deceitful, patronising, dignity-reducing to have dolls and allow people to believe they are real babies.	*Offering aspects of legacy* *Acknowledging the person behind the patient* *Facilitating meaningful engagement*
15. Higgins P. (2010) Using dolls to enhance the wellbeing of people with dementia in residential care, Nursing Times, 106 (39): 18–20 [65]	Doll therapy	- Theoretical - Literature review on how dolls can enhance wellbeing of people with dementia in residential care.	*Offering aspects of legacy* *Acknowledging the person behind the patient* *Facilitating meaningful engagement*
16. Holm A., Lepp M. and Ringsberg KC. (2005) Dementia: involving patients in storytelling- a caring intervention. A pilot study, Journal of Clinical Nursing, 14, 256–263 [101]	Storytelling	- Empirical - Pilot study exploring the therapeutic role of storytelling in patients with dementia. - Participants were 6 patients (5 women, 1 man), who had intermediate and severe dementia. Three female paid caregivers also participated. - Participants met on 6 occasions within 2 months. Each meeting involved participants gathering in a circle and being told a story by the leader.	*Offering aspects of legacy* *Facilitating meaningful engagement*
17. Ingersoll-Dayton B., Spencer B., Kwak M., Scherrer K., Allen RS., and Campbell R. (2013) The Couples Life Story Approach: A Dyadic Intervention for Dementia, Journal of Gerontological Social Work, 56: 3, 237–254 [79]	- Couples Life Story Approach (adapted from Legacy Therapy); Life story work; Reminiscence - Usually involves one-hour sessions over five weeks	- Empirical - Couples (of which one partner has dementia), reminisce about their relationship story using photographs and mementoes (postcards, newspaper clippings, wedding vows) and develop a book - Intervention engages both care recipient and caregiver, and endeavours to focus on meaningful engagement and shared communication - Final study sample couples (*n* = 20); Study conducted in couples’ homes; family member home; care retirement community	*Acknowledging the person behind the patient* *Offering aspects of legacy* *Facilitating meaningful engagement*
18. Kasl-Godley J. and Gatz M. (2000) Psychosocial interventions for individuals with dementia: an integration of theory, therapy, and a clinical understanding of dementia [18]	Various including focus on Reminiscence and Life Review	- Theoretical - integrative review on six psychosocial interventions for individuals with dementia. - Interventions described in terms of theoretical underpinnings, techniques and relatable empirical evidence.	*Offering aspects of legacy* *Acknowledging the person behind the patient*
19. McDermott O., Orrell M. and Ridder HM. (2014) The importance of music for people with dementia: the perspectives of people with dementia, family carers, staff and music therapists, Aging & Mental Health, 18(6): 706–716 [89]	Music-based interventions	- Empirical - Qualitative study exploring the importance and meaning of musical experiences for people with dementia - Focus groups and interviews conducted with care home residents with dementia and their families; day hospital clients with dementia; care home staff; music therapists - Residents from 2 NHS care homes (Home A- *N* = 45; Home B- *N* = 24); Staff were those who provide day-to-day care to residents; Family members were those who had significant contact with residents	*Facilitating meaningful engagement* *Offering aspects of legacy* *Acknowledging the person behind the patient*
20. McKeown J., Clarke A., Ingleton C., Ryan T. and Repper J. (2010) The use of life story work with people with dementia to enhance person-centred care, International Journal of Older People Nursing, 5, 148–158 [72]	Life story work	- Empirical - Multiple case study design used, including interviews, observations and conversations with older people with dementia (*n* = 4), family carers and care staff within an NHS Health and Social Care Trust. - Focuses on how life story work enhances person-centred care for individuals with dementia	*Offering aspects of legacy* *Acknowledging the person behind the patient* *Facilitating meaningful engagement*
21. McKeown J., Clarke A. and Repper J. (2006) Life story work in health and social care: systematic literature review, Journal of Advanced Nursing, 55(2): 237–247 [85]	Life story work (LSW)	- Theoretical - systematic literature review on life story work in health and social care practice - Staff views frequently explored, but sparse evidence around patient and carer perceptions.	*Offering aspects of legacy* *Facilitating meaningful engagement* *Acknowledging the person behind the patient*
22. McKeown J.,Ryan T., Ingleton C., Clarke A. (2015) “You have to be mindful of whose story it is”: The challenges of undertaking life story work with people with dementia and their family carers, Dementia, 14(2): 238–256 [81]	Life story work	- Empirical - Case study analysis to gain insight into experiences of using life story work in one NHS Mental Health and Social Care Trust (across four different NHS care settings). - Participants were people with dementia (*n* = 4), family carers and care staff - Data collection comprised of semi-structured interviews, observations, conversations and field notes	*Offering aspects of legacy* *Acknowledging the person behind the patient*
23. Moos I. and Björn A. (2006) Use of life story in the institutional care of people with dementia: a review of intervention studies, Ageing and Society, 26, 431–454 (come back to) [93]	Life story; Reminiscence	- Theoretical - review of 28 intervention studies that endeavoured to explore the benefits of life story for nursing home residents with dementia (in particular looking at links to residents’ sense of identity). - Papers published between 1990 and 2003 - Interventions were divided into 3 groups: Interventions to raise self-esteem and self-integration; Interventions to change life quality; Interventions to change behaviour	*Acknowledging the person behind the patient* *Facilitating meaningful engagement* *Offering aspects of legacy*
24. Pringle A. and Somerville S. (2013) Computer-assisted reminiscence therapy, Mental Health Practice, 17(4): 34–37 [86]	Reminiscence therapy (computer-assisted)	- Empirical - Describes the early development stages of a pilot study looking at using new technology in reminiscence therapy in for people with dementia in inpatient settings (*n* = 8). - This involves a tablet device containing a reminiscence file for each patient. The files may encompass photographs, films, song playlists and music. - Three sessions were carried out, led by a member of staff. The 1^st^ session used structured conversation, 2^nd^ used a memory book; 3^rd^ used the computer tablet only	*Offering aspects of legacy*
25. Russell C. and Timmons S. (2009) Life story work and nursing home residents with dementia, Nursing Older People, 21(4): 28–32 [94]	Life story work	- Empirical - Using narrative research methodology, the stories of 5 nursing home residents with dementia were analytically reconstructed. - Participants were over the age of 65 years and had mild to moderate symptoms of dementia. - Unstructured interviews were used, which were recorded and transcribed verbatim with ideas also written down.	*Offering aspects of legacy* *Acknowledging the person behind the patient*
26. Savundranayagam MY., Dilley LJ. and Basting A. (2011) StoryCorps’ Memory Loss Initiative: Enhancing personhood for storytellers with memory loss, Dementia, 10(3): 415–433 [62]	Life story work	- Empirical - Study around the American StoryCorps’ Memory Loss Initiative for collecting oral histories of people with memory loss. - Each conversation told through StoryCorps is recorded and produced on a broadcast-quality CD. This is archived at the Library of Congress, following participants’ permission. - Investigates to interview experience (specifically follow-up interviews) of people with memory loss (*n* = 42) and their family members (*n* = 27) In Chicago and New York, America.	*Facilitating meaningful engagement* *Acknowledging the person behind the patient* *Offering aspects of legacy*
27. Scherrer KS., Ingersoll-Dayton B. and Spencer B. (2013) Constructing Couples’ Stories: Narrative Practice Insights from a Dyadic Dementia Intervention, Clinical Social Work Journal, 42, 90–100 [96]	Couples Life Story Approach	- Empirical - Exploring the effects of a 5 week structured dyadic intervention to provide couples with a chance for meaningful engagement, to explore their strengths, to enhance communication and to encourage them to reflect on their shared experiences. - Sample was 20 couples (40 individuals), one of whom had memory loss.	*Acknowledging the person behind the patient* *Offering aspects of legacy*
28. Subramaniam P., Woods B. and Whitaker C. (2013) Life review and life story books for people with mild to moderate dementia: a randomised controlled trial, Aging & Mental Health, 18 (3): 362–375 [95]	Life review	- Empirical - Evaluation of the effect of different pathways for producing a life story book (LSB) for people with dementia. - Participants were 23 people with dementia in care homes - RCT with two intervention arms: 1) 12 individual life review sessions and co-creating a LSB; 2) A personal LSB created by their relatives as a “gift”. - Results suggested no significant difference in quality of life between the two groups six weeks after the LSB had been received (*F*(1,20) = 0.08, *p* = 0.77). But quality of life had improved for both groups. - There was significant between-group difference immediately after the life review sessions had been carried out but before the LSBs were received (F(1, 20) = 5.11, *p* = 0.035). - Regardless of pathway, production of LSBs led to improved quality of relationships (rated by relatives) (F(2, 39) = 19.37, *p* < 0.001).	*Offering aspects of legacy* *Acknowledging the person behind the patient*
29. Thompson R. (2011) Using Life Story Work to enhance care, Nursing Older People, 23(8): 16- 21 [54]	Life Story Work	- Theoretical - literature review on the notion of life story work and tools used to elicit information about the person. - Benefits for people with dementia, family members and staff are highlighted. - Barriers are also acknowledged, and include lack of time, support, resources, skills and confidence.	*Offering aspects of legacy* *Acknowledging the person behind the patient*
30. Williams BR., Blizard TI., Goode PS., et al. (2014) Exploring the affective dimension of the life review process: Facilitators’ interactional strategies for fostering personhood and social value among older adults with early dementia, Dementia, 13(4): 498–524 [80]	Life review	- Empirical - Study based on individual one-on-one conversational sessions with community-dwelling military veterans (*n* = 12) with Mild Cognitive Impairment (MCI) and early dementia. - A life review workbook was used to support the conversations, which had been produced by the Hospice Foundation of America. - Participants had two to four life review sessions, which were recorded. Informal caregivers could be present. - Each session was a maximum of 2 h. - Interviews were conducted in a private office in the veterans’ “Medical Center” (*n* = 10), or in the veterans’ place of residence (*n* = 2)	*Offering aspects of legacy* *Acknowledging the person behind the patient* *Facilitating meaningful engagement*
31. Alzheimer Scotland- Action on Dementia (2014) Annual review 2013–14, The Scottish Government [75]	Personalised profile forms- “Getting to know me”	- Grey literature - Discusses the development and use of the “Getting to know me” form by NHS Lanarkshire - Insight from a Dementia Nurse Consultant working for NHS Lanarkshire, to explain how it is used in practice	*Offering aspects of legacy* *Acknowledging the person behind the patient*
32. Alzheimer Scotland- Action on Dementia (2013) Dementia in Scotland, Winter 2012/13, Issue 77 [76]	Personalised profile forms- “Getting to know me”	- Grey literature - Discusses the use of personalised profile forms within NHS Lanarkshire- “Getting to know me”. - Used by Band 6 nurses (Charge Nurses and Deputy Charge Nurses) to look at their strategies for improving the experiences of people with dementia and their families in hospitals. - Explores how using “Getting to know me” in practice has informed nurses’ care and allowed them to use the information to use strategies for dealing with difficult situation	*Offering aspects of legacy* *Acknowledging the person behind the patient*
33. Health Improvement Scotland (2012) Announced Inspection Report- care for older people in acute hospitals- Hairmyres Hospital, NHS Lanarkshire, Scotland: Health Improvement Scotland [77]	Personalised profile forms- “Getting to know me”	- Grey literature - Reports on an announced inspection looking at the care of older people in acute hospitals - Highlights that NHS Lanarkshire is piloting (at the time of publishing) the “Getting to know me” document.	*Offering aspects of legacy* *Acknowledging the person behind the patient*
34. Kane, M. (2012) My life until the end- Dying well with dementia, Alzheimer’s Society [74]	Personalised profile forms – “This is me”	- Grey literature - Report exploring seven key issues that need to be taken account for people with dementia at end of life: Public awareness; Care planning and proxy decision making; Dignity; Pain; Withholding and withdrawing treatment; Emotional and spiritual concerns; Place of care and death - The report is informed by semi-structured interviews with former carers (*n* = 25), current carers (*n* = 10), and people with dementia (*n* = 3). - Further insight was provided by Alzheimer’s Society colleagues working with people with dementia.	*Acknowledging the person behind the patient*
35. Robinson P. and Tyndale-Biscoe J. (2014) What makes a top hospital? Dementia care- report 7, Warwickshire: Caspe Healthcare Knowledge Systems (CHKS) [73]	Personalised profile forms- “This is me”	- Grey literature - Report outlining recommendations for hospitals to enable them to deliver better care for people with dementia - Endorses the use of “This is me” document, which was developed by the Northumberland Acute Care and Dementia Group with support from the Royal College of Nursing. - Although initially developed for people with dementia going into hospital, it is appropriate for use in any setting where professional care is being received.	*Acknowledging the person behind the patient* *Facilitating meaningful engagement*
36. Royal College of Nursing (RCN) (2013) Dementia- Commitment to the care of people with dementia in hospital settings, London: RCN [55]	Personalised profile forms- “This is me”	- Grey literature - Resource providing guidance to people working in hospital settings to help them to deliver high quality care for people with dementia and their carers. - Includes brief discussion of “This is me”, and considers it a version of life story work.	*Offering aspects of legacy* *Acknowledging the person behind the patient*

### Offering aspects of legacy

The identified psychosocial interventions that directly allowed people with dementia to leave a legacy were life story work and reminiscence therapy. In most cases, this materialised as a tangible document, such as, a life story book, which contained photographs, text, postcards, letters and memorabilia [18].

Moreover, there were also variations such as memory boxes [52], video biographies [61], multimedia biographies and CD recordings [62–64]. Memory boxes allowed meaningful objects to be contained beyond photographs, for example a smoking pipe [52]. Hagens et al. [52] study involved reminiscence sessions with five older adults who had cognitive impairment. Participants’ words and phrases were incorporated into a personal poem. In addition, Remembering Boxes were also produced to include meaningful objects and writings. The poems and Remembering Boxes came to be person-centred communication tools since they helped staff to learn more about residents, serving to be particularly useful when the latter were sleepless or agitated. The poems were framed and were placed in the residents’ rooms along with the Remembering Boxes. Both tangible objects were found to provide staff with insights into the residents and encouraged staff to take further interest in the resident. Moreover, in contrast with books and boxes, some tangible objects did not always convey an obvious biographical narrative. For example, interventions such as doll therapy [57, 65], animal therapy [66], place therapy [67] and object-stimulated reminiscence therapy [68–70], served to enable reminiscence of earlier life experiences, as opposed to conveying experiences immediately, as with a book. The legacy component could be inferred in the fact that the resulting reminiscing allowed insight into people’s identity and selves, which had been unknown previously to family members and healthcare professionals [52, 54, 57, 64, 71]. In some cases, knowledge of the person’s life story gave caregivers guidance to implement person-centred care and helped them to understand reasons behind people’s behaviour and learn what was important to them in the present [71, 72].

Furthermore, the grey literature provided insight into how life story work has been adapted for different healthcare settings. This has led to personalised profile form versions such as “This is me” [55, 73, 74] and “Getting to know me” [75–77]. These personalised forms allow people with dementia (often on admission to hospital settings) to provide a snapshot of themselves by inserting brief information in response to questions on the forms. An RCN resource [55] highlighted “This is me” as a version of the life story books that usually materialise from life reviews, thus making a link to an established, evidence-based body of work. This RCN resource suggests that personalised profile forms may be more amenable to hospital settings compared to life story books, due to their brief form and the fact that they are quick to fill in. However, there was also recognition that such forms should be appropriate for use in any setting where professional care is being received [73]. The grey literature confirmed that such personalised profile forms were present in patient health records, which highlights that they may be applicable to clinical practice. [77]. Moreover, one source recommended personalised profile forms to be used in practice in all settings catering for people with dementia, as a way of promoting dignity [74]. The legacy component for such forms can be seen as preserving the personal preferences of patients as individuals and letting these be known to people caring for them.

Where there was a legacy component inherent in the intervention, this was referred to in some of the studies [18, 62, 64, 78–80] and the intervention was commended for allowing the person’s life story to be in a form that could endure, be revisited, prevail and be accessed by future generations within and out of the family. This was largely the case for life story work interventions, where in most cases, a life story book, in some form or another, is produced. Cohen’s [61] literature review described life story products, such as video biographies, as an exit gift. Savundranayagam et al. [62] empirically explored a life story initiative known as StoryCorps, which aims to preserve a record of the life stories of people with memory loss by means of broadcast CDs. Some family members declared that they would save listening to the CD for when their loved one died. Moreover, family members often saw this strategy as a way of allowing the person with memory loss to be heard, which may or may not routinely happen in care or in wider society. Ingersoll-Dayton et al. [79] explored couples’ life stories as adapted from legacy therapy. One partner in each couple had dementia, and were encouraged to reminisce about their relationship using photographs and mementoes. As a collaborative process, the intervention was found to engage both partners and is designed to focus on meaningful engagement and shared communication. However, the research sample used for this study is somewhat limited, since it consisted of white, heterosexual couples who lived in their homes.

Likewise, Cohen’s [61] (2002) literature review exploring non-medical interventions to bring about patient satisfaction, suggested that life story work and video biographies could be an intergenerational project, which involves younger family members. This positive aspect of life story work was also recognised in another study [18]. Cohen [61] provides a strong rationale advocating for the use of video biographies, describing them as therapeutic and restorative. They suggest that they can assist family and friends with sharing time with the person with dementia. This is particularly relevant when dementia conditions, such as Alzheimer’s Disease can be dehumanising, and prevents the person from relating their personal histories. This would suggest that video biographies allow a legacy to be passed on while the person with dementia is still alive. Nevertheless, Cohen’s review only looks at one study around video biographies.

In McKeown et al. [72] life story work was not framed as a “couple” intervention, it was still carried out as a collaborative process, but prioritised the person with dementia’s choices as to what went into the life story book. On the other hand, this was not always the case, since one study [81] found that the wife of one man with dementia chose not to include particular photographs in her husband’s life story book. This raises the question of whether it is truly the person with dementia’s story that is being presented and preserved, which then has implications for the legacy component of this intervention. Moreover, Chung [78] looked at an intergenerational reminiscence programme between older participants with dementia, and youth volunteers. The programme involved reminiscence sessions and the production of life story book. Although, findings suggested that there were mutual benefits for both participants, this was based mostly on feedback from youth participants rather than the older people. Therefore, more detailed analysis around the advantages for older participants would be helpful in order to ascertain how useful and/or relevant legacy aspects are for the person with dementia. In addition, quality of life story books were not always of a high standard, as one study [81] reported that errors were present including typing, spelling and grammatical mistakes, as well as pictures not being secured adequately.

With couple life story work, the resulting life story book means that couples have something to review and revisit and many couples spoke about intentions to share the book with others [79]. McKeown et al. [72] also found that the revelations of life histories that came from life story work intervention allowed the person behind the patient to emerge, and enabled staff to learn new things, which helped them to understand the person better. Cohen’s [61] literature review suggests that such enhanced knowledge acquired by staff can increase their sensitivity.

However, despite being perceived as a positive intervention, there were practical difficulties, such as, struggles to find pictures for the book [79]. Moreover, for some, it was a bittersweet experience to revisit the past and was seen to be preferable earlier on in the illness trajectory. Further criticism for this intervention was found in an integrative review [18] which suggested that although life story work and reminiscence can allow a legacy to be left and fulfil interpersonal functions [82–84], for individuals who have difficulties in processing past experiences, leaving a legacy may prove problematic. Another, systematic review [85] focusing on life story work suggested that patient and carer perceptions were less likely to be explored compared to staff. Further criticism suggests that life story work was time consuming from staff perspectives [62, 85]. Nevertheless, life story books were seen to have potential to contribute to regular assessment documentation to enable new information about the person to be conveyed [72]. This suggests that the legacy component of life story work is not just relevant after death, but also during the person with dementia’s life, with life story books offering a way to enhance person-centred care, and preserve personhood before illness deterioration. In addition, staff value life story work as a way of allowing patients to be seen as people with histories, and endorse this intervention for helping them to gain insight into present behaviours by learning about the person’s past [72].

Other variations of the life story book exist, such as personal computer files [86] and CDs [62]. These digital versions allowed more sophisticated content, such as music playlists, and allowed a more seamless process [86]. There were also other objects beyond books which were recognised as having legacy components. Buse and Twigg [69] discussed the use of clothing items, specifically, handbags, in triggering reminiscence of personal memories. This was motivated by the notion that handbags are linked to memories and identities that enable females to reacquaint them with positive periods of their life, such as, motherhood. Aspects of legacy were present since handbags were described as biographical objects, serving to facilitate storytelling and disclosures of personal histories. Handbags were identified as prevailing objects, which are retained when illness disrupts a person’s biography. [87] Specifically, while some husbands of women with dementia could not understand why their wives wanted to keep their collections of handbags, which were seemingly functionless in the current context, other relatives could see how handbags allowed the retention of the person who was otherwise lost through illness or death, emphasising personhood.

The production of DVD-based Multimedia Biographies were also explored at in two related studies [63, 64], which were based on empirical work with participants with Alzheimer’s Disease. Production of Multimedia Biographies were a collaborative process between people with dementia and their families and content included photographs, home movies, documents, music and narration. As well as, providing a tangible product that allowed personal experiences and stories to be recorded and kept, it also helped family members to conserve their loved one’s personhood, providing a means to convey the person’s story to future generations. Family members credited the Multimedia Biographies with facilitating intergenerational communication and enabling a family legacy to be left; specifically, patient participants valued being able to leave a legacy for their loved ones after they had died [88]. However, the authors [63] also note that it should be established whether or not the person with dementia wants to share the Multimedia Biographies, or whether they would prefer to view them alone. In all circumstances, the person’s wishes regarding this should be adhered to. Nevertheless, the authors do, however, highlight that producing Multimedia Biographies can be complex because of the complicated technology and time required.

Conversely, some studies did not explicitly discuss the interventions as relating to legacy. For instance, Benbow [68] discussed a literature review focusing on four studies, which looked at designing residential environments to facilitate meaningful activities. The article mainly discussed the aims and objectives to be achieved by incorporating designs such as “lifestyle stations” into the residence of people with dementia. This included the intention to allow people with dementia to access memories of previous hobbies and/or working life, resulting in the production of vignettes that enable residents to recognise particular activities and practice specific skills as derived from their life stories. The review identified studies which showed that lifestyle stations and other designs, such as photographic memory triggers and technology, stimulated reminiscence about former roles and helped retrieve residents’ meaning of self and purpose. In addition, in their qualitative study exploring the importance and meaning of musical experiences for people with dementia, McDermott et al. [89] elicited perspectives on music-based interventions. Participants included residents with dementia from two care homes with dementia, care home staff and music therapists. Music was perceived to be emotionally meaningful for people with dementia and allowed various levels of engagement including listening to music and singing. Staff acknowledged that music triggers particular memories, which then leads to notable positive changes in residents. Individuals were described as having a musical identity, which that related to specific life events and eras. Although, lyrics and songs can have a legacy component, particularly in terms of memories, this was not highlighted in the study. In addition, the authors of this paper acknowledge that their findings may not be adequately representative of people with mild to moderate dementia.

Similarly, Chaudhury [67] conducted an empirical study of residents from nursing homes which included 13 people with dementia, who were encouraged to disclose their narratives of lived experiences as stimulated by meaningful places from their past. Childhood places in particular were well recalled and associated life events were also accessed. Photographs served to enhance access to such narratives. The process was recognised to promote the person’s sense of self. Places were specifically recognised as significant for offering a way to structure meanings of the person’s past. Therefore, aspects of legacy here can be seen to be inherent in the existence of the place themselves, and also the aids (e.g., photographic aids) that allow recall of such places. Photographic aids were also used to encourage personal storytelling in another study [90]. Similarly, Gibb et al. [70] analysed the stories elicited from nine people with dementia, who underwent paired and group reminiscence sessions following Tai Chi exercises. The Tai Chi exercises were seen to aid focusing thinking. The initial reminiscence procedure, which involved cognitive and psychomotor tasks were eventually deemed inappropriate for most participants, so incorporated tangible cues, e.g., guided imagery and photo albums, from the fourth session onwards. The tangible cues were not discussed as having legacy attributes, but did help participants to divulge past events and treasured memories. These were particularly centred on early life, including, parenting and family. However, participants were unable to follow their narrative through to a full construction conveying life experience.

Other studies where the aspects of legacy were not made explicit, but were surmised, included those where there was not a tangible outcome; rather knowledge and insight into the person with dementia was gained. For instance, a grounded theory study by Cooney et al. [71] used in-depth interviews with residents with dementia, their relatives, and healthcare professionals to gain insight into their perceptions of reminiscence. The main implications were at a practical level, in which staff were able to ascertain what was important for the residents in the present, by learning about their past. Therefore, the legacy component was acquired through knowledge, as opposed to a tangible life story book. However, conversations were boosted through the use of photographs to encourage disclosures. The authors do caution that the success of implementing reminiscence is affected by the resident’s stage of dementia, co-morbidities and personal preferences; staff time availability; and the organisational culture of long stay facilities. This study was mainly drawn from data relating staff perspectives, which limits understanding into residents’ points of view, and as such, less person centred.

A literature review looking at the benefits of pet therapy [66] suggested that the presence of an animal can stimulate particular memories and conversations around these memories, including talking about past pet ownership. Discussed in the context of nursing homes, the author does, however, highlight particular cautions. These include an awareness that pets may not be welcomed in a communal space; particular animals may be regarded as “unclean” or “dangerous” by some cultural groups; and the presence of an animal may pose potential risks to some residents. Just as the presence of a pet was identified as triggering specific memories, doll therapy was also identified as a psychosocial intervention used to gain insight into people with dementia [57]. In their review, Heathcote and Clare [57] suggested that dolls had symbolic significance, and allowed people to convey feelings that they struggled to communicate to others. In addition, they found that interacting with dolls can help people with dementia to think about the past and make sense. However, these authors also discuss the possible controversies of this type of intervention, which may have ethical implications [57]. These include issues around whether doll therapy is deceitful, in the case of allowing the person with dementia to believe that the doll is a baby or living being; whether it is patronising; and questions over whether the person’s dignity is reduced through use of the intervention.

In addition, another literature review [65] highlights that empirical evidence for the use of dolls is sparse and much of the information is provided by anecdotal sources. Nevertheless, these anecdotal evidence remain positive for the use of these tangible dolls [65]. In contrast to the criticism of doll therapy being dignity-reducing, Higgins’ review [65] argues that it does in fact preserve dignity, since the intervention can allow someone with dementia to take on a familiar role, which may have been rewarding for them earlier in life. Moreover, Heathcote and Clare’s review [57] suggests that other similar objects could be used to facilitate reminiscence that may not be perceived as controversial, such as toy trains [91].

Therefore, with much of the literature, even though legacy was not directly mentioned, it could be surmised from the nature of the intervention, that they had the potential for legacy. This was the case for articles discussing life story work, reminiscence therapy, music therapy using song lyrics, doll therapy and animal therapy; where there was something tangible that could be linked to memories and disclosed life stories [52, 54, 66, 69, 70, 72–75, 79, 81, 86, 89, 90, 92–95]. Although, it is possible to see the legacy potential in the fact that tangible memories were posited, this was not acknowledged within these sources and was not a recognised aim or focus of the research. This does suggest that the legacy components of these psychosocial interventions would be worthy of more explicit exploration in the research evidence.

Many of the studies and reports that were reviewed discussed life story work and reminiscence and resulting life story books as positive, by highlighting that such approaches allowed aspects of the person’s identity to come to the fore, which was emphasised with particular prominence in the grey literature [74–76]. However, as mentioned before, the literature also identified drawbacks relating to ownership and influence of content of life story books [81], where family members sometimes had priority over the person with dementia as to what was included. This meant that some aspects of the person’s life could not be represented, and as such was less person centred.

### Acknowledging the person behind the patient

Some psychosocial interventions allowed the person’s identity to be emphasised beyond their illness. This included ways in which the interventions enabled former roles, experiences and achievements to be revisited. Psychosocial interventions which included some form of reminiscence were more likely to allow people with dementia to explore previously held roles, enabling part of the person’s life story to be conveyed. Many of the interventions allowed people with dementia to revisit past roles through memories or activities. Access to past roles through memories was the case for studies and research around reminiscence and life story work [64, 80, 95]. According to these studies, triggering previous feelings based on earlier experiences was generally found to be positive for the person. Moreover, where there was a tangible product stemming from the intervention, relatives also appeared to benefit from being given the opportunity to learn about the person. Scherrer et al. [96] study on couples’ life story work revealed that the partner of Matthew- a man with Alzheimer’s Disease- wanted his time in employment to be recognised during the sessions and in the resulting life story book. This was shown to bring about excitement for Matthew, suggesting that it was an important time in his life. The actual dyadic intervention was, however, designed to bring about memories and insights into their life as a couple, as opposed to separate memories.

In a narrative research study conducted with five nursing home residents with mild to moderate dementia [94], life story work was found to show the individuality of each participant by acknowledging that they each had a different story to tell, motivated by different reasons. These involved recalling former roles and making sense of traumatic events. Whilst McKeown et al. [85] agree with the positive effects of life story work, acknowledging that it can be successful in preserving memories of earlier experiences and roles, they warn that it may also lead to thoughts about loss resulting from the illness.

The review found that particular objects were also found to stimulate reminiscence, whilst serving to provide insight into previously held roles, for example lifestyle stations [68], handbags [69] and memory boxes [52]. Buse and Twigg’s [69] study, in particular, drew attention to the pertinence of handbags as biographical objects with specific links to identity, for example larger bags being associated with parenthood and carrying children’s belongings. Moreover, although content such as money had no function in the present context of where the female participants in this study were, money was symbolic as it had once been very significant and linked to previous roles and responsibilities, such as shopping for the family. Indeed, the authors suggest that discarding handbags for these women may symbolise resignation to institutional life.

In Benbow’s [68] study looking at design features, the four studies featured in the review discussed how designs triggered reminiscence by purposely building on residents’ life stories and former roles/skills. For example memory stations encourage residents to practice particular skills that are derived from their life stories, including former job roles. In addition, Damianakis et al. [64] looking at Multimedia Biographies from life story work intervention found that the content helped to trigger previous feelings that patients had about themselves, based on points in their earlier lives. This included what they were wearing and who they were with in photographs. This also proved beneficial for family members who had forgotten what the person with dementia had been like pre-illness. Similarly, in Subramaniam et al. [95] evaluation of life review and life story book production, one son of a participant with dementia, appreciated the chance to revisit memories and life events that he had shared with his mother. Remembering Boxes created from reminiscence sessions also allowed objects to be accommodated, which represented past interests and roles [52].

Beyond reminiscence and life story work, doll therapy was also highlighted as a means of allowing former roles and aspects of identity to be brought to the fore. Doll therapy was endorsed as an intervention to help people with dementia to take on familiar roles [57, 65]. Higgins [65] exploration of doll therapy cites Gibson’s [97] study in which the daughter of a lady with dementia found that having a doll allowed the latter to access a time in her life where she felt in control. Moreover, it allowed the daughter to draw comfort by considering that this display of love and affection would have been applied to her when she was a baby. Doll therapy may be particularly helpful for people who have an inherent maternal and/or nurturing desire and may lead to reminiscing about their role as parents as found with Healthcote and Clare’s study [57] and cited studies within Heathcote and Clare’s review [98, 99]. Similarly, animal assisted therapy was found to trigger conversations recounting memories of being a pet owner [66]. However, as addressed earlier, there are controversies which exist around the use of both doll therapy and animal-assisted therapy. Doll therapy may be met with negative reactions [65] and instigate ethical dilemmas as to whether or not such therapy is patronising, undignified and operates by deception since some people with dementia may believe the doll to be a real baby [57]. Animal-assisted therapy may be difficult to implement in communal settings such as nursing and residential homes since not all residents may like animals, based on their personal and/or cultural beliefs [66].

Music-based interventions were also found to help acknowledge the person behind the patient, as explored through qualitative interviews and focus groups with residents with dementia in care homes, their families, care home staff and music therapists [89]. One resident spoke about singing music he remembers from being a child, despite forgetting other things. However, reminiscing about previous roles through the use of music was also upsetting, as it reminded one of past youth, which is now gone. This is a reminder of McKeown et al. work on life story interventions, which also highlighted this issue [72, 85].

The theme of *Acknowledging the person behind the patient* was also apparent in situations where the intervention helped the person with dementia to convey and express aspects of their present identity. Retaining aspects of self, identity and individuality was evident in studies around reminiscence therapy, with this particular intervention endorsed as a means of focusing on the person’s ability as opposed to their impairment [52, 100]. Likewise, Williams et al. [80] report found that the life review process allowed an outlet for the complex inner worlds of people with dementia, thus reflecting great self-awareness. Life story work and reminiscence was found to generate a sense of self, enhance self-esteem, help self-understanding and affirm selfhood ([18, 62–64, 93]. One study [62] highlighted how life story work served to re-affirm the selfhood of participants who had memory loss and re-establish their relationships with family members. Likewise, family members were encouraged to see the person who is still present. Also of significance is the way in which life story work can help establish a person’s future by clarifying wishes [54], whilst also showing the diversity of identity by revealing unique stories for each person [94].

Specific interventions were salient in helping to promote identity of people with dementia. For instance, Buse and Twigg [69] found that handbags were a key object representing normalcy for female residents in care homes. When looking at place-based reminiscence, Chaudhury [67] found that induced home-related memories contributed to continuation of the self. The doll therapy could provide opportunities for people with dementia to fulfil a natural maternal instinct and thus extending previously held roles into the present [65]. Similarly, McDermott et al. [89] pick up on “musical” identities that emerge through the experience of engaging with music. The literature also identified studies which took into account the partners of people with dementia and reported the benefits of couples’ life story approaches [79, 96]. This technique allowed communication and understanding between couples to improve and also recognised the status of the person with dementia as being part of a couple.

Subscribing to our perspective of legacy as stated earlier, *Acknowledging the person behind the patient* can be seen to link with legacy, since it encourages and helps the person with dementia to reveal more about their story in terms of their identity and achievements.

Grey literature sources, which explored the use of personalised profile forms, highlighted that these forms enable staff to have a clearer picture of present issues for the patient, in terms of their likes, dislikes, preferences, and key information such as important relationships. As key facets of identity, making a record of these aspects can provide insight into the person behind the patient. The literature acknowledges that personalised profile forms were seen to enhance person-centred care and promote dignity [55, 73, 74, 76, 77].

### Facilitating meaningful engagement

This theme contributed to understandings of how the intervention enhanced personhood, because it identified ways in which the person with dementia elicited feelings of meaning and purpose. In the studies reviewed, this often translated as whether the person enjoyed the experience of the intervention and gained pleasure. These aspects were well explored in the studies, presented as specific aims as part of analysing effects of the interventions.

Subsequent analysis and discussion in several studies acknowledged whether the intervention allowed meaningful engagement and enjoyment to materialise. The insight provided by *Facilitating meaningful engagement* is particularly useful when using this review’s earlier definition of “personhood” as referring to the status of being a person and related elements such as recognition, respect and trust. Meaningful engagement was often linked to the interventions’ ability to instigate hope. Interventions that facilitated meaningful engagement were either directly meaningful or enabled meaningful activities to follow [62, 65, 67, 68, 80, 89]. For instance, Holm et al. [101] looked at the therapeutic role of storytelling in patients with dementia. Participants attended a group session in which they were encouraged to take part in associative conversations through storytelling and bring in past experiences. The intervention led to participants feeling pleasure and experiencing fellowship. Reminiscence provided hope by enabling the person with dementia to be treated as an individual. This was particularly noticeable for Cooney et al. study [71] featuring care home residents, who were encouraged by the fact that staff were interested in their lives [71]. Moreover, staff specifically noted that the residents’ engagement improved and that relationship between staff and residents were strengthened. Being listened to was also acknowledged as significant to participants who had undergone a life review in Williams et al. [80] study. In addition, Moos and Björn [93], highlight a number of studies which found that wellbeing, enjoyment and interactions increased following individual and group reminiscence [102–106]. Musical interventions also served to be emotionally meaningful for participants who had late-stage dementia in McDermott et al. study [89], with one participant finding that songs helped to support his personal identity.

The production of Remembering Boxes and poetry, following reminiscence sessions in Hagens et al. study [52], led to joyful moments for residents with dementia, particularly facilitating stimulating conversations and positive feelings. Moreover, the residents featured in this study were able to take control and lead interactions with others, which gave them satisfaction, wand also motivated further conversation. The Remembering Boxes underwent testing to confirm that they did not lead to anxiety or agitation. In addition, Dempsey et al. [100] concept analysis into reminiscence suggested that the intervention can aid problem solving in the present situation by guiding the person to draw on past coping strategies. This can help the person to acquire a sense of continuity and meaning in life. However, these authors also raise awareness about the potential negative effects regarding the retrieval of unhappy memories around loss or pain. They concede that negative repercussions can be guarded against if a person-centred approach to care is taken, including gaining knowledge of the person [107]. One study [62] explored a life story programme which collected the oral histories of people with memory loss and presented these in a high-quality broadcast CD. Participants with memory loss implied that one positive aspect was that it allowed access to memories that they did not realise they had.

Secondary outcomes of using life story work and reminiscence were often focused on enhanced connections and stimulating interactions with others such as relatives [52, 64]. Moreover, this was particularly enhancing to personhood when the person with dementia was found to be leading and controlling such interactions [52]. In addition, specifically life story work was found to help participants with dementia to find meaning in loss and enabled reflection and engagement [62, 80, 100]. Prominent authorship of life story products, such as Multimedia Biographies in Crete-Nishihata et al. study [63] meant that participants experienced self-growth and a sense of having achieved something. With couple’s life story work [79], the couples were reported to have enjoyed the collaborative process and this was partly reflected in increased intimacy. Moreover, allowing the couple to review their life together in this way emphasised the partnership, and acknowledged the meaningful relationship that they have together. Similarly, storytelling as an intervention offered meaning to life by bringing consolation to participants [101].

A systematic review looking at life story work [85] suggested that patients who received the intervention were more likely to describe it as an enjoyable activity. Furthermore, this was not just due to the activity per se, but also because of elements such as companionship, which was achieved through the sharing of the book. Benefits of life story work were also apparent in the grey literature. Robinson and Tyndale-Biscoe [73] report that a personalised profile form, developed for people with dementia to fill out on admission to hospital, has the potential to improve communication for patients. On the other hand, a one study with older people with dementia [72] suggested that participants did not explicitly show that they enjoyed the life story intervention, but conceded that they did convey pride at showing their life story and receiving interest in this.

One empirical study looked specifically at life review [80]. Life reviews are sessions which encourage a person to recount and evaluate through their life experiences chronologically, and may result in the production of a life story book [108]. Williams et al. [80] used one-on-one conversational sessions with community-dwelling military veterans who had mild cognitive impairment and early dementia. These authors found that even when thinking about dissatisfaction and past regrets, the life review helped participants to find meaning in loss and thus served to conserve their dignity. The life review process also allowed participants to consider strengths and weaknesses, and therefore be more accepting of circumstances. In addition, being listened to (as facilitated through the life review process) was met with positive reactions by participants and enhanced their personhood.

## Discussion

### Legacy and personhood

Understanding the extent to which psychosocial interventions were related to legacy was best understood through the themes of *Offering aspects of legacy* and *Acknowledging the person behind the patient.* Largely, psychosocial interventions which related to legacy were some form of either life story work or reminiscence therapy. Understanding a person’s biography is highlighted as an important aspect of person-centred care [43, 72]. Moreover, it is recognised that humans are narrative beings [109–111], and one’s story is pertinent to identity and the self [112]. This is supported by the idea that illness has been described as a biographical disruption [113]. In this review, some studies mentioned the legacy aspects and highlighted that this allowed the person’s story to be captured in a form that could endure, be revisited, prevail and be accessed by future generations within, and out, of the family. However, generally, the legacy aspect of interventions was not explicitly explored or developed, and when legacy potential existed, it was not always highlighted; rather it was inferred by us, as the authors of this review, based on our earlier established definition of legacy.

Life story work interventions in the studies reviewed, allowed participants to convey their stories, and in many cases, the stories were recorded through life story books or digital formats. The adaptation of this to personalised profile forms for hospital environments were only identified through largely grey literature. Such forms have been described as a “personal passport”, enabling a person’s personal history to be captured and to help get a sense of the person beyond the illness [114]. Reminiscence was highlighted as having different intentions to Life Story Work [18, 100], with the latter going beyond recall of memories and incorporating evaluation and re-synthesis of past experiences. The studies around reminiscence discussed the use of objects to stimulate recall and reveal life stories. They allowed former key roles to be revisited. For example, doll therapy [57, 65] and animal therapy [66] enabled the nurturing role of early parenthood to be fulfilled. Similarly, Buse and Twigg’s [69] study showed how handbags were significant biographical objects, which could stimulate reminiscence about being a parent or dressing up for an evening out. Although, Benbow’s [68] review around design features (such as the implementation of lifestyle stations), clearly demonstrated how specific former roles could influence design, the effect of this was not developed, due to a lack of empirical data. Another intervention that had legacy potential was storytelling [90] which utilised general and personal photographs to revelations of stories.

The two themes of *Offering aspects of legacy* and *Acknowledging the person behind the* patient, also allowed the psychosocial interventions to be assessed for their ability to enhance personhood. This is because acknowledging the identity of the person beyond the illness, attributes a human status to them. Interventions with a legacy component serve to help preserve key aspects of the person’s identity from past and present and can enable these to prevail beyond illness deterioration and death.

However, when relating more specifically to enabling meaning and purpose, assessing personhood was best done through the theme of *Facilitating meaningful engagement.* Personhood attributes the status of being a person to an individual [43] and has gained pertinence in research concerning people with dementia, since people with dementia are thought to be particularly vulnerable to having personhood eroded [44]. Dewing [44] proposes that this is linked to the cultural belief that associates intact cognition with the status of being a person. Moreover, personhood is linked to personal continuity and the continuity of one’s narrative [115, 116]. Personhood is also linked to hope and existential reflections around meaning and purpose [45, 117]. The studies in this review generally explored personhood aspects well, particularly in terms of meaning, purpose, and enhanced wellbeing and engagement. The studies conveyed aspects of personhood as aims of the research and explored how these aspects of personhood were impacted by the interventions through analysis. The enablement of individuals to maintain their self and identity, elicit hope and enjoyment, and find meaning and hope, were well reported in the studies, with empirical studies highlighting evidence around levels of enjoyment, accommodating discussion around people’s verbal and non-verbal reactions to interventions. In addition, specifically the intervention(s) enabling a sense of continuity for the person were also identified. For example, notions were generated about how interventions enabled people to foster a sense of self and allowed abilities to be recognised. However, some literature only discussed the intervention(s) as having the potential to provide such benefits, as opposed to citing empirical evidence [67, 68].

### Strength and limitations

This integrative review has had a particularly focused agenda to assess psychosocial interventions based on their link to personhood and legacy and has recognised themes according to these. This could potentially mean that other important issues and themes, unrelated to personhood and legacy, may be key to understanding the perspectives of people with dementia, and these have not have been considered or highlighted. For example, past reviews have looked at effects of psychosocial interventions in reducing symptoms of dementia. This review had a focused aim to explore personhood and legacy, so only focused on these aspects during analysis. Although, aspects of personhood were explored in much of the studies included, the term “personhood” was not always mentioned directly. It is possible that our conceptualisation of personhood, though based on established definitions, may not resonate with other people’s understandings. Methodological limitations exist around the processes of inclusion and exclusion, which were based on what may be considered as subjective criteria. Part of the exclusion process was based on whether the intervention had the potential to enhance personhood and enable the person to leave a legacy. In some cases, this was difficult to establish from the title and abstract, and may have meant that relevant articles were excluded. Moreover, the inability to perform methodological appraisal on such diverse sources of literature, means that articles could not be assessed for methodological strengths. This means that applicability to practice may be limited, since this review cannot ensure that it offers findings from the most methodologically strong papers.

However, despite these limitations, this review has addressed an under-researched area, which is particularly important in terms of understanding the perspectives of people with dementia. Findings indicate that legacy components and enhancement of personhood are important and relevant to people with dementia, their families and staff involved in their care, suggesting that further research will be beneficial. To address the gap in this area, the review incorporated sources from across the evidence base to offer a comprehensive integrative review of all available research evidence, and gives a useful overview of which psychosocial interventions relate to personhood and legacy, how they do this, and what some of the effects are.

### Implications for practice care and research

Aspects of personhood were generally well reported in the studies included. Although personhood was not always specifically termed, based on the definitions that this review subscribed to, it is evident that enhancement of personhood has been explored as an effect of interventions with some positive effects acknowledged. This suggests that formal and informal carers of people with dementia could consider life story work, reminiscence therapy, doll therapy and animal assisted therapy as strategies to possibly enhance personhood. Specifically with life story work, which also offers a legacy component, the review has found that people with dementia value this when a collaborative element is present. It would be useful for “personhood” as a concept to be directly addressed and explored in research. However, there are limitations to these interventions and caution erred that should be taken into account when using these for people with dementia. Such limitations have been briefly outlined in this review, for example, the fact that life story work may bring about feelings of loss [85]; reminiscence leading to the retrieval of unpleasant memories [100]; the controversies around doll therapy [57, 65]; and the difficulties that may be faced with implementing animal-assisted therapy [66].

With regards to assessing the interventions, based on their relation to legacy much had to be surmised, since, although, the potential for legacy could be identified and we were able to recognise ways in which the interventions offered aspects of legacy, the articles themselves did not highlight this or adequately develop exploration around this. Moreover, legacy potential was never prioritised as part of the research agenda. In order to ascertain helpful insights into interventions which relate to legacy, more focused studies on this aspect are needed. In particular, and in line with established definitions of “legacy”, it would be useful to see whether legacy products such as life story books are read by families and future generations after the person with dementia has died. It may be particularly useful to directly explore the perspectives of people with dementia to ascertain whether they feel that legacy components are important for available psychosocial interventions.

## Conclusion

This integrative review had a focused agenda to identify, appraise and assess interventions for people with dementia, as described in the literature, based on their ability to enhance personhood and relate to legacy. This has been achieved by identifying key themes to help classify previous research around this and acknowledge the potential for further research. Generally, although personhood aspects were well reported, insights into legacy requires further attention and needs to be looked at more specifically and even beyond the patient’s life trajectory. Therefore, further research might involve making exploration of legacy components a key, prioritised aim of the research. In addition, more perspectives from people with dementia are needed, rather than family members and staff.

## Data availability statement

All data are in the paper and supplementary files.

## References

[1] Society A’s (2015). Types of dementia [cited.

[2] World Health Organization. Dementia 2015 [cited 2015 March]. Available from: http://www.who.int/mediacentre/factsheets/fs362/en/. Accessed 30 Sept 2015.

[3] Harris D (2007). Forget me not: palliative care for people with dementia. Postgrad Med J.

[4] Scottish Government. Scotland’s National Dementia Strategy: 2013–16. Edinburgh: Scottish Government; 2014.

[5] Department of Health (2013). Dementia- a state of the nation report on dementia care and support in England. Health.

[6] Kenigsberg P-A, Aquino J-P, Be’rard A, Gzil F (2015). Dementia beyond 2025: knowledge and uncertainties.

[7] Larson EB, Yaffe K, Langa KM (2013). New insights into the dementia epidemic. N Engl J Med.

[8] Ouldred E, Bryant C (2009). A practical guide to dementia. Br J Healthc Manag.

[9] Society A’s (2015). Alzheimer’s Society and Public Health England launch Dementia Friends TV campaign 2014 [cited 2015 March].

[10] Ballard C. Editorial. Alzheimer’s Society [Internet]. 2010; (11):[2 p.]. Available from: http://www.alzheimers.org.uk/site/scripts/download_info.php?fileID=1001. Accessed 30 Sept 2015.

[11] Ballard C. Which activities are most engaging for people with dementia living in care homes? 2010; Alzheimer’s Society(11). Available from: http://www.alzheimers.org.uk/site/scripts/download_info.php?fileID=1001. Accessed 30 Sept 2015.

[12] Nazarko L (2009). Dementia care: the use and abuse of anti-psychotic drugs. Nurs Residential Care.

[13] Ballard CG, Gauthier S, Cummings JL, Brodaty H, Grossberg GT, Robert P (2009). Management of agitation and aggression associated with Alzheimer disease. Nat Rev Neurol.

[14] Hungerford C, Jones T, Cleary M (2014). Pharmacological versus nonpharmacological approaches to managing challenging behaviours for people with dementia. Br J Community Nurs.

[15] World Health Organization (WHO). Dementia- A Public Health Priority. United Kingdom; 2012.

[16] Hulme C, Wright J, Crocker T, Oluboyede Y, House A (2010). Non‐pharmacological approaches for dementia that informal carers might try or access: a systematic review. International journal of geriatric psychiatry.

[17] National Institute for Health and Clinical Excellence (NICE)/ Social Care Institute for Excellence (SCIE). Supporting people with dementia and their carers in health and social care. London; 2006.

[18] Kasl-Godley J, Gatz M (2000). Psychosocial interventions for individuals with dementia: an integration of theory, therapy, and a clinical understanding of dementia. Clin Psychol Rev.

[19] Patel B, Perera M, Pendleton J, Richman A, Majumdar B. Psychosocial interventions for dementia: from evidence to practice. Advances in psychiatric treatment. 2014;20(5):340–9

[20] Selwood A, Johnston K, Katona C, Lyketsos C, Livingston G (2007). Systematic review of the effect of psychological interventions on family caregivers of people with dementia. J Affect Disord.

[21] Thompson CA, Spilsbury K, Hall J, Birks Y, Barnes C, Adamson J. Systematic review of information and support interventions for caregivers of people with dementia. BMC Geriatr. 2007;7(18). 10.1186/1471-2318-7-18 PMC1951962 17662119

[22] Brodaty H, Green A, Koschera A (2003). Meta-analysis of psychosocial interventions for caregivers of people with dementia. J Am Geriatr Soc.

[23] Cooke DD, McNally L, Mulligan KT, Harrison MJG, Newman SP (2001). Psychosocial interventions for caregivers of people with dementia: a systematic review. Aging Ment Health.

[24] Pusey H, Richards D (2001). A systematic review of the effectiveness of psychosocial interventions for carers of people with dementia. Aging Ment Health.

[25] Regan B, Varanelli L (2013). Adjustment, depression, and anxiety in mild cognitive impairment and early dementia: a systematic review of psychological intervention studies. Int Psychogeriatr.

[26] O’Connor DW, Ames D, Gardner B, King M (2009). Psychosocial treatments of psychological symptoms in dementia: a systematic review of reports meeting quality standards. Int Psychogeriatr.

[27] Livingston G, Johnston K, Katona C, Paton J, Lyketsos CG (2005). Systematic review of psychological approaches to the management of neuropsychiatric symptoms of dementia. Am J Psychiatry.

[28] Testad I, Corbett A, Aarsland D, Lexlow KO, Fossey J, Woods B (2014). The value of personalized psychosocial interventions to address behavioral and psychological symptoms in people with dementia living in care home settings: a systematic review. Int Psychogeriatr.

[29] Moniz-Cook E. Psychosocial interventions for ‘living well with dementia’ in care homes. Alzheimers Soc Res e-J. [Internet]. 2010;(11). Available from: http://www.alzheimers.org.uk/site/scripts/download_info.php?fileID=1001. Accessed 30 Sept 2015.

[30] Vernooij-Dassen M, Vasse E, Zuidema S, Cohen-Mansfield J, Moyle W (2010). Psychosocial interventions for dementia patients in long-term care. Int Psychogeriatr.

[31] Robinson L, Hutchings D, Dickinson HO, Corner L, Beyer F, Finch T (2007). Effectiveness and acceptability of non-pharmacological interventions to reduce wandering in dementia: a systematic review. Int J Geriatr Psychiatry.

[32] Yuhas N, McGowan B, Fontaine T (2006). Interventions for disruptive symptoms of dementia. J Psychosoc Nurs Ment Health Serv.

[33] Richter T, Meyer G, Mohler R, Kopke S (2012). Psychosocial interventions for reducing antipsychotic medication in care home residents. Cochrane Database Syst Rev [Internet].

[34] Carrion C, Aymerich M, Baillés E, López-Bermejo A (2013). Cognitive psychosocial intervention in dementia: a systematic review. Dement Geriatr Cogn Disord.

[35] Spector A, Woods B, Orrell M (2008). Cognitive stimulation for the treatment of Alzheimer’s disease. Expert Rev.

[36] Van Mierlo LD, Van der Roest HG, Meiland FJM, Dröes RM (2010). Personalized dementia care: proven effectiveness of psychosocial interventions in subgroups. Ageing Res Rev.

[37] Lawrence V, Fossey J, Ballard C, Moniz-Cook E, Murray J (2012). Improving quality of life for people with dementia in care homes: making psychosocial interventions work. Br J Psychiatry.

[38] Bates J, Boote J, Beverley C (2004). Psychosocial interventions for people with a milder dementing illness: a systematic review. J Adv Nurs.

[39] Johnston B, Pringle J, Gaffney M, Narayanasamy M, McGuire M, Buchanan D. The dignified approach to care: a pilot study using the patient dignity question as an intervention to enhance dignity and person-centred care for people with palliative care needs in the acute hospital setting. BMC palliative care. 2015;14(1):1. 10.1186/s12904-015-0013-3 PMC4399754 25883533

[40] Love K, Pinkowitz J (2013). Person-centred care for people with dementia: a theoretical and conceptual framework. J Am Soc Aging.

[41] Chochinov H (2007). Dignity and the essence of medicine: the A, B, C, and D of dignity conserving care. BMJ.

[42] Gallagher A, Li S, Wainwright P, Jones IR, Lee D (2008). Dignity in the care of older people: a review of the theoretical and empirical literature. BMC Nurs.

[43] Kitwood T (1997). The experience of dementia. Aging Ment Health.

[44] Dewing J (2008). Personhood and dementia: revisiting Tom Kitwood’s ideas. Int J Older People Nurs.

[45] Social Care Institute for Excellence (SCIE) (2013). Dementia gateway: knowing the person behind the dementia.

[46] Stein-Parbury J, Chenoweth L, Jeon YH, Brodaty H, Haas M, Norman R (2012). Implementing person-centered care in residential dementia care. Clin Gerontol.

[47] Twigg J, Buse CE (2013). Dress, dementia and the embodiment of identity. Dementia.

[48] World Health Organization. Ensuring a human rights-based approach for people living with dementia. Geneva: 2015.

[49] Society A’s (2015). Factsheet: end-of-life care [cited 2015 March].

[50] Dictionary.com. legacy [cited 2015 March]. Available from: http://dictionary.reference.com/browse/legacy. Accessed 30 Sept 2015.

[51] Dementia UK (2015). Life story work [cited 2015 March].

[52] Hagens C, Beaman A, Ryan EB (2003). Reminiscing, poetry writing, and remembering boxes: personhood-centered communication with cognitively impaired older adults. Act Adapt Aging.

[53] Chochinov HM (2012). Dignity therapy: final words for final days.

[54] Thompson R (2011). Using life story work to enhance care. Nurs Older People.

[55] Royal College of Nursing (RCN). Dementia- Commitment to the care of people with dementia in hospital settings. London: 2013.

[56] Attachment BJ (1969). Attachment and loss.

[57] Heathcote J, Clare M (2014). Doll therapy: therapeutic or childish and inappropriate?. Nurs Residential Care.

[58] Nelson-Jones R (2000). Six key approaches to counselling & therapy.

[59] Warren B (2008). Using the creative arts in therapy and healthcare: a practical introduction.

[60] Torraco RJ (2005). Writing integrative literature reviews: guidelines and examples. Hum Resour Dev Rev.

[61] Cohen GD (2002). Creating a video biography for a loved one who has memory impairment. Geriatrics.

[62] Savundranayagam MY, Dilley LJ, Basting A (2011). StoryCorps’ memory loss initiative: enhancing personhood for storytellers with memory loss. Dementia.

[63] Crete-Nishihata M, Baecker RM, Massimi M, Ptak D, Campigotto R, Kaufman LD (2012). Reconstructing the past: personal memory technologies are not just personal and not just for memory. Hum Comput Interact.

[64] Damianakis T, Crete-Nishihata M, Smith KL, Baecker RM, Marziali E (2009). The psychosocial impacts of multimedia biographies on persons with cognitive impairments. Gerontologist.

[65] Higgins P (2010). Using dolls to enhance the wellbeing of people with dementia in residential care. Nurs Times.

[66] Heathcote J (2010). Paws for thought: involving animals in care. Nurs Residential Care.

[67] Chaudhury H (2003). Quality of life and place-therapy. J Hous Elder.

[68] Benbow B (2014). Design features for resident engagement and meaningful activity. Can Nursing Home.

[69] Buse C, Twigg J (2014). Women with dementia and their handbags: negotiating identity, privacy and ‘home’ through material culture. J Aging Stud.

[70] Gibb H, Morris CT, Gleisberg J (1997). A therapeutic programme for people with dementia. Int J Nurs Pract.

[71] Cooney A, Hunter A, Murphy K, Casey D, Devane D, Smyth S (2014). ‘Seeing me through my memories’: a grounded theory study on using reminiscence with people with dementia living in long-term care. J Clin Nurs.

[72] McKeown J, Clarke A, Ingleton C, Ryan T, Repper J (2010). The use of life story work with people with dementia to enhance person-centred care. Int J Older People Nurs.

[73] Robinson P, Tyndale-Biscoe J. What makes a top hospital? Warwickshire: 2014.

[74] Kane M (2012). My life until the end- dying well with dementia.

[75] Alzheimer Scotland. Alzheimer Scotland dementia nurse network. Edinburgh: 2014.

[76] Scotland A (2013). Dementia in Scotland.

[77] Health Improvement Scotland (2012). Announced inspection report- care for older people in acute hospitals.

[78] Chung JC (2009). An intergenerational reminiscence programme for older adults with early dementia and youth volunteers: values and challenges. Scand J Caring Sci.

[79] Ingersoll-Dayton B, Spencer B, Kwak M, Scherrer K, Allen RS, Campbell R (2013). The couples life story approach: a dyadic intervention for dementia. J Gerontol Soc Work.

[80] Williams BR, Blizard TI, Goode PS, Harada CN, Woodby LL, Burgio KL (2014). Exploring the affective dimension of the life review process: facilitators’ interactional strategies for fostering personhood and social value among older adults with early dementia. Dementia.

[81] McKeown J (2015). You have to be mindful of whose story it is’: the challenges of undertaking life story work with people with dementia and their family carers. Dementia.

[82] Molinari V, Reichlin RE. Life review reminiscence in the elderly: a review of the literature. Int J Aging Hum Dev. 1984–1985;20(2):81–92. 10.2190/k4mg-9vyg-wql3-cbrh 6396235

[83] Romaniuk M (1981). Reminiscence and the second half of life. Exp Aging Res.

[84] Romaniuk M, Romaniuk JG (1981). Looking back: an analysis of reminiscence functions and trigger. Exp Aging Res.

[85] McKeown J, Clarke A, Repper J (2006). Life story work in health and social care: systematic literature review. J Adv Nurs.

[86] Pringle A, Somerville S (2013). Computer-assisted reminiscence therapy: developing practice. Ment Health Pract.

[87] Woodward S (2007). Why women wear what they wear.

[88] Smith KL, Crete-Nishihata M, Damianakis T, Baecker RM, Marziali E (2009). Multimedia biographies: a reminiscence and social stimulus tool for persons with cognitive impairment. J Technol Hum Serv.

[89] McDermott O, Orrell M, Ridder HM (2014). The importance of music for people with dementia: the perspectives of people with dementia, family carers, staff and music therapists. Aging Ment Health.

[90] Fels DI, Astell AJ (2011). Storytelling as a model of conversation for people with dementia and caregivers. Am J Alzheimers Dis Other Demen.

[91] Cayton H, Hughes JC, Louw SJ, Sabat SR (2006). From childhood to childhood? Autonomy and dependence through the ages of life. Dementia, mind, meaning and the person.

[92] Holm A-K, Lepp M, Ringsberg KC, Sellersjo G (2003). Dementia - involving patients and their caregivers in a drama programme: the caregivers’ experiences. J Clin Nurs.

[93] Moos I, Bjorn A (2006). Use of the life story in the institutional care of people with dementia: a review of intervention studies. Ageing Soc.

[94] Russell C, Timmons S (2009). Life story work and nursing home residents with dementia. Nurs Older People.

[95] Subramaniam P, Woods B, Whitaker C (2013). Life review and life story books for people with mild to moderate dementia: a randomised controlled trial. Aging Ment Health.

[96] Scherrer KS, Ingersoll-Dayton B, Spencer B (2013). Constructing couples’ stories: narrative practice insights from a dyadic dementia intervention. Clin Soc Work J.

[97] Gibson S (2005). A personal experience of successful doll therapy. J Dementia Care.

[98] Moore D (2001). ‘It’s like a gold medal and it’s mine’ - dolls in dementia care. J Dementia Care.

[99] Minshull K (2009). The impact of doll therapy on well-being of people with dementia. J Dementia Care.

[100] Dempsey L, Murphy K, Cooney A, Casey D, O’Shea E, Devane D (2014). Reminiscence in dementia: a concept analysis. Dementia.

[101] Holm AK, Lepp M, Ringsberg KC (2005). Dementia: involving patients in storytelling--a caring intervention. A pilot study. J Clin Nurs.

[102] Brooker D, Duce L (2000). Wellbeing and activity in dementia: a comparison of group reminiscence therapy, structured goal-directed group activity and unstructured time. Aging Mental Health.

[103] Gibson F, Bornat J (1994). What can reminiscence contribute to people with dementia?. Reminiscence reviewed: evaluation, achievements, perspectives.

[104] Götell E, Brown S, Ekman SL (2000). Caregiver-assisted music events in psychogeriatric care. J Psychiatr Ment Health Nurs.

[105] Olsen RV, Hutchings BL, Ehrenkrantz E (2000). ‘Media memory lane’: interventions in an Alzheimer’s day-care center. Am J Alzheimers Dis.

[106] Rentz CA (2002). Memories in the making: outcome-based evaluation of an art program for individuals with dementing illnesses. Am J Alzheimers Dis Other Dement.

[107] Swee H, Heathcote J (2005). Part one: the value of reminiscence. Nurs Residential Care.

[108] Woods B, Spector AE, Jones CA, Orrell M, Davies SP. Reminiscence therapy for dementia. Cochrane Database Syst Rev. 2005;2005(2). 10.1002/14651858.CD001120.pub2 15846613

[109] Williams S, Keady J (2006). Editorial: the narrative voice of people with dementia. Dementia.

[110] Baldwin C (2005). Narrative, ethics and people with severe mental illness. Aust N Z J Psychiatry.

[111] Bruner J (1987). Life as narrative. Soc Res: An Int Q.

[112] Kelly MP, Field D. Medical sociology, chronic illness and the body. Sociol Health Illn. 1996;18(241–57).

[113] Bury MR (1982). Chronic illness as biographical disruption. Sociol Health Illn.

[114] Charalambous L (2015). Comment: “About me” puts a person at the heart of patient-centred care. Nurs Times.

[115] Harre R (1998). The singular self: an introduction to the psychology of personhood.

[116] Apter M, Breakwell G (1989). Negativism and the sense of identity. Threatened identities.

[117] Allen FB, Coleman PG, Hughes JC, Louw SJ, Sabat SR (2006). Spiritual perspecives on the person with dementia: identity and personhood. Dementia mind, meaning and the person.

